# Effectiveness of Tai Chi on Physical and Psychological Health of College Students: Results of a Randomized Controlled Trial

**DOI:** 10.1371/journal.pone.0132605

**Published:** 2015-07-06

**Authors:** Guohua Zheng, Xiulu Lan, Moyi Li, Kun Ling, Hui Lin, Lidian Chen, Jing Tao, Junzhe Li, Xin Zheng, Bai Chen, Qianying Fang

**Affiliations:** 1 College of Rehabilitation Medicine, Fujian University of Traditional Chinese Medicine, Fuzhou 350122, China; 2 Department of Physical Education, Fujian University of Traditional Chinese Medicine, Fuzhou 350122, China; 3 Fujian University of Traditional Chinese Medicine, Fuzhou 350122, China; Hunter College, UNITED STATES

## Abstract

**Objective:**

To investigate the effectiveness and safety of Tai Chi Chuan (TCC) on physical and psychological health of college students.

**Methods:**

Two hundred six college students were recruited and randomly allocated to a control group or a TCC exercise group in an equal ratio. Participants in the control group were instructed to maintain their original activity level and those in the TCC exercise group received 12 weeks of TCC exercise training based on their original activity level. Physical and psychological outcomes were evaluated at baseline, 13 weeks and 25 weeks. Intention-to-treat analysis was performed for the above outcomes.

**Results:**

Compared with the control group, the TCC exercise group showed significant improvements at the end of the 12-week intervention period for flexibility (length of Sit and Reach (cm): TCC group 14.09±7.40 versus control 12.88±6.57, *P* = 0.039 adjusted for its baseline measures using a general linear model) and balance ability (open eyes perimeter: TCC group 235.6(191~314) versus control 261(216~300); closed eyes perimeter: TCC group 370.5 (284~454) versus control 367 (293~483); *P* = 0.0414, 0.008, respectively, adjusted for corresponding baseline measures using a general linear model). No significant changes in other physical and mental outcomes were found between the two groups. No adverse events were reported during the study period.

**Conclusion:**

TCC exercise was beneficial in college students for improving flexibility and balance capability to some extent, compared with usual exercise.

**Trial Registration:**

Chinese Clinical Trial Registry ChiCTR-TRC-13003328

## Introduction

College life is a critical period during which individuals begin to take definitive steps toward independence and is also considered to be the first major transition period of growth and development that bridges adolescence and adulthood [1]. This period usually involves many rapid changes in the body, mind, and social relationships. The lifestyle and behaviors that an individual develops during this stage may remain into adulthood and impact future health status. However, with the gradual increase of competitive pressure among students, the level of physical activity and exercise in college students has declined worldwide [2–4]. According to the National College Health Assessment (U.S.A), less than 60% of college students achieve the minimal recommendation of 30 minutes per day of moderately intense physical activity [5]. The percentage of physically inactive college students was 13.5% in Taiwan, 16.8% in Hong Kong, and 28.5% in Korea [6]. Physical inactivity is known to increase risk of cardiovascular disease and even all-cause mortality [7–8]. Many studies have demonstrated that college students might be a population at risk for and susceptible to chronic diseases, including obesity, metabolic syndrome, hypertension, and diabetes [9–11]. In addition, the psychological/mental well-being of college students might be ‘worse off’ than that of the general population. College students were more likely to suffer from various forms of mental health problems than same-aged non-student populations due to multiple extra stressors, such as academic challenges, competition and achievement [11–15]. A survey of 14,175 college students in the United States showed that the prevalence of psychobiological problems was 17.3% for depression, 4.1% for panic disorder, 7.0% for generalized anxiety, 6.3% for suicidal ideation, and 15.3% for non-suicidal self-injury [16]. If left ignored and untreated, mental health problems could lead to students dropping out of college, attempting or committing suicide, or engaging in other dangerous behaviors [17]. Furthermore, it is estimated that only a minority of college students with mental health problems seek and receive adequate help [18].

Growing research continues to strengthen the idea that regular exercise or physical activity is positively associated with physical and psychological health outcomes [19–20]. Tai Chi Chuan (TCC) and Baduanjin are two of the most common traditional Chinese mind-body exercises, and have been practiced to promote the holistic health of general population for many centuries in China [21–22]. Although both TCC and Baduanjin are low-impact fitness exercises that rooted in the traditional Chinese medicine theory. Unlike TCC, Baduanjin exercise is characterized by simple, slow, relaxing movements and composed of 8 set of sitting and standing posters [21]. While the classical TCC consisted of complex forms (e.g. Chen style consists of 108 posters), and need long time to learn and practice. In the process of development, many simplified TCC styles have developed to shorten the learning period (e.g. the simplified Yang style only consists of 24 posters). TCC is preformed in a semi-squat position with continuous, cured, and spiral body movement [23]. Through deep diaphragmatic breathing and basic slow and gentle mind-body movements, practitioners can achieve the effect of ‘body relaxation and mind calm’ and Tian Ren He Yi (the theory that mankind is an integral part of nature) [22]. TCC and Baduanjin are ideal mind-body exercises for people of different ages with different physical and health conditions, especially for those who live a sedentary lifestyle and who lack the motivation to engage in more conventional exercise [21–24]. Several systematic reviews have suggested that TCC or Baduanjin may be effective in promoting physical and psychological health in the middle-aged and elderly populations [25–30]. However, to date, no study of high methodological quality has been conducted to investigate whether TCC or Baduanjin can be recommended as an effective exercise for improving the emotional state, psychological well-being and physical fitness of young adults, particularly the college student population. Therefore, we designed two rigorous, prospective randomized controlled trials to further investigate the effects of TCC or Baduanjin as an intervention for specific psychological outcomes in a college student population [31–32]. Here we reported the effects of TCC for college students.

## Methods/Design

### Study Design

The protocol for this trial has been previously published [32], and supporting CONSORT checklist is available as S1 File. This study was a two-arm, randomized, parallel controlled trial in college students, conducted in China. Participants were recruited between September 1^st^ and September 30^th^ of 2013, with data collected between October 2013 and March 2014. The trial was registered with Chinese Clinical Trial Registry in May 2013 (ChiCTR-TRC-13003328; http://www.chictr.org.cn/).

### Ethical Consideration

This study was approved by the ethics committee of the Fujian University of Traditional Chinese Medicine (2013, Reviewed-No.042). All eligible participants who consented to study participation were over 18 years of age and provided written consent. All participants had the right to withdraw from the study at any time.

### Participant Recruitment

Participants were recruited from the Fujian University of Traditional Chinese Medicine (FJTCM) using advertisements on the campus bulletin board and on campus radio. Eligible criteria included: age 16 to 25 years, freshman or sophomore enrolled in full-time study, and provision of informed consent. Excluded criteria included: had engaged in a long-term TCC exercising or other Tai Chi derived movements, or a member of the Students' Wushu Association, Taekwondo Association, Aerobic Association or Dance Association, or suffered from severe cardiovascular disease or musculoskeletal disease. After provided signed, written consent, eligible participants were randomized to the TCC exercise group or control group. The random allocation sequence was generated by a statistician not involved in the study, using the Statistical Analysis System (SAS, version 9.1). The allocation sequence was concealed with password access files and was kept with the project manager. After the baseline assessment, the project manager informed each eligible participant of his/her allocation. It is not possible to blind participants and TCC instructors to the group allocation in this trial. We assigned a project manager to manage the randomized allocation sequence and to blind the allocation code, in which the TCC exercise group and control group were renamed A or B. All data were collected by research assistants or professional staff in the hospital blinded to group allocation. The project manager and the TCC instructors were not involved in the assessment of outcomes and data analyzers were not involved in the recruitment and allocation of participants. The blinded allocation sequence codes were built to blind the data analyzer and, thus, were not disclosed until statistical analysis had been completed.

### Tai Chi Chuan Exercise Intervention

The program of TCC exercise training consisted of 60 minutes exercise sessions, five days per week for 12 weeks, based on their original level of physical activity. Each session comprised 40 minutes of TCC training plus a 10-minute warm-up and cool-down. The TCC training was instructed by two experienced TCC instructors who were qualified and engaged in the teaching of TCC for at least 15 years. The 24 forms of simplified TCC recommended as the popular health sport by the General Administration of Sport of China [33–34] were applied. Five sessions per week were performed at the campus gymnasium from 5:20 p.m. to 6.20 p.m. The intervention period lasted 12 weeks from October 15, 2013 to January 5, 2014. Adherence was monitored by the instructor, and attendance was managed by project manager through the attendance record form. In addition, participants were required to record a daily diary of physical activity, including type and intensity of physical activity or exercise, sedentary time and sleep time.

### Control Group

No specific exercise intervention was administered to participants in the control group. They were informed to maintain their usual physical activities. The daily diary of physical activities was also required to be recorded.

### Follow-Up Period

An unsupervised 12-week follow-up period for participants began on January 6, 2014 and ended on March 30, 2014. No participants received additional exercise intervention, but participants were required to record their daily activity in a log.

### Outcome Measurement

Demographic characteristics were obtained from an interviewer administered questionnaire at recruiting. The primary outcome measures were changes in balance ability, lower limb proprioception, and flexibility, as well as self-reported psychological symptoms, self-efficiency, stress, and attention post-intervention. We also evaluated the longer-term effects over a 12-week follow-up period at week 25 (first week after completion of the 12-week follow-up period). Balance ability and lower limb proprioception function were tested by blinded operators from the Evaluation Department of the Rehabilitation Hospital Affiliated with FJTCM using the Pro-kin system (Machine type: produced by *Italy Tecnobody*. *S*.*R*.*L company*, *model PK254P*) [35–37]. Flexibility was tested using Sit and Reach flexibility test equipment (Machine type: *Beijing ZhongTi Tongfang Co*., *Ltd*., *model CSTF-TQ-5000*). Scores for self-efficiency, psychological symptoms, stress and attention were assessed using their corresponding scales of confirmed validity [38–40].

Secondary outcomes included changes in cardio-pulmonary function, blood pressure, heart rate, mood and mindfulness, self-esteem, quality of life, and quality of sleep at 13 weeks (first week after completion of the 12-week intervention period) and at 25 weeks (first week after completion of the 12-week follow-up period). Cardio-pulmonary function, consisting of cardiopulmonary fitness and vital capacity, was evaluated using an electronic step test instrument (stairs with step height of 30 centimeters for males and step height of 25 centimeters for females) (Machine type: *Beijing ZhongTi Tongfang Co*., *Ltd*., *model CSTF-TZ-5000*) and an electronic vital capacity instrument (Machine type: *Beijing ZhongTi Tongfang Co*., *Ltd*., *model CSTF-FH-5000*). Blood pressure and resting heart rate were measured by electric sphygmomanometers produced by the *Omron Corporation*, *China* (product type: *HEM-746C*). Self-esteem, mood and mindfulness, quality of life, and quality of sleep were assessed using corresponding self-reported questionnaires [41–42]. Quality of life was tested with the *WHOQOL-BREF scale* [43]. The Chinese versions of these scales have been reported to have acceptable internal consistency, test-retest reliability, construct validity, and criterion-related validity. Details about the outcome assessments were described in the published protocol [32].

### Safety Evaluation

Any adverse event was recorded on a case report form (CRF) during the intervention period.

### Statistical Analysis

The primary and secondary outcomes were analyzed with an intention-to-treat (ITT) basis. The missing data were imputed by using Fully Conditional Specification (FCS) algorithm of multiple imputations which is an iterative Markov Chain Monte Carlo (MCMC) method. Most of primary or secondary variables (i.e. open eyes perimeter, open eyes ellipse area, close eyes perimeter, close eyes ellipse area, right lower limb ATE, left lower limb ATE, self-efficacy, psychological symptom, stress, attention, self-esteem, quality of life, quality of sleep, SBP, DBP, rest heart rate, vital capacity, and flexibility) measured at baseline, end of 12 week intervention, or end of 12 week follow-up period were included in the imputation model. Five complete datasets were created, and each dataset was performed the ITT analysis and then the results were summarized by using Rubin’s rules [44]. The continuous variables were expressed as means with standard deviations for normal distributions and medians with inter-quartile ranges for non-normal distributions. Categorical variables were expressed as proportions with standard errors. Comparison of baseline characteristics between groups was conducted using a *t*-test or non-parametric test for continuous variables and the Pearson chi-squared or Fisher exact test for categorical variables. Comparison of primary and secondary outcomes between groups from baseline measurements was performed using a general linear model with adjustment for possible confounders.

All statistical analyses were performed using IBM SPSS 21.0 (version 21.0, IBM Corp., New York, NY, USA) software package. A two-sided *p* value of less than or equal to 0.05 was considered to be statistically significant.

### Sample Size

We estimated a sample size of 103 participants per group (206 in total) was necessary to detect a expected difference of 15% in one of primary outcomes (balance ability, represented by motion of ellipse area which was tested by standing with eyes closed on the flat of Pro-kin system) between the TCC exercise and control group, with assuming a type I error of 5% (α = 0.05), 90% power (β = 0.10) and allowing for 10% dropout rate. The 15% expected difference in means between groups and standard deviations were estimated from our preliminary test data. Details about the sample size estimated were also described in the published protocol [28].

## Results

### Baseline Characteristics

**Fig 1:**
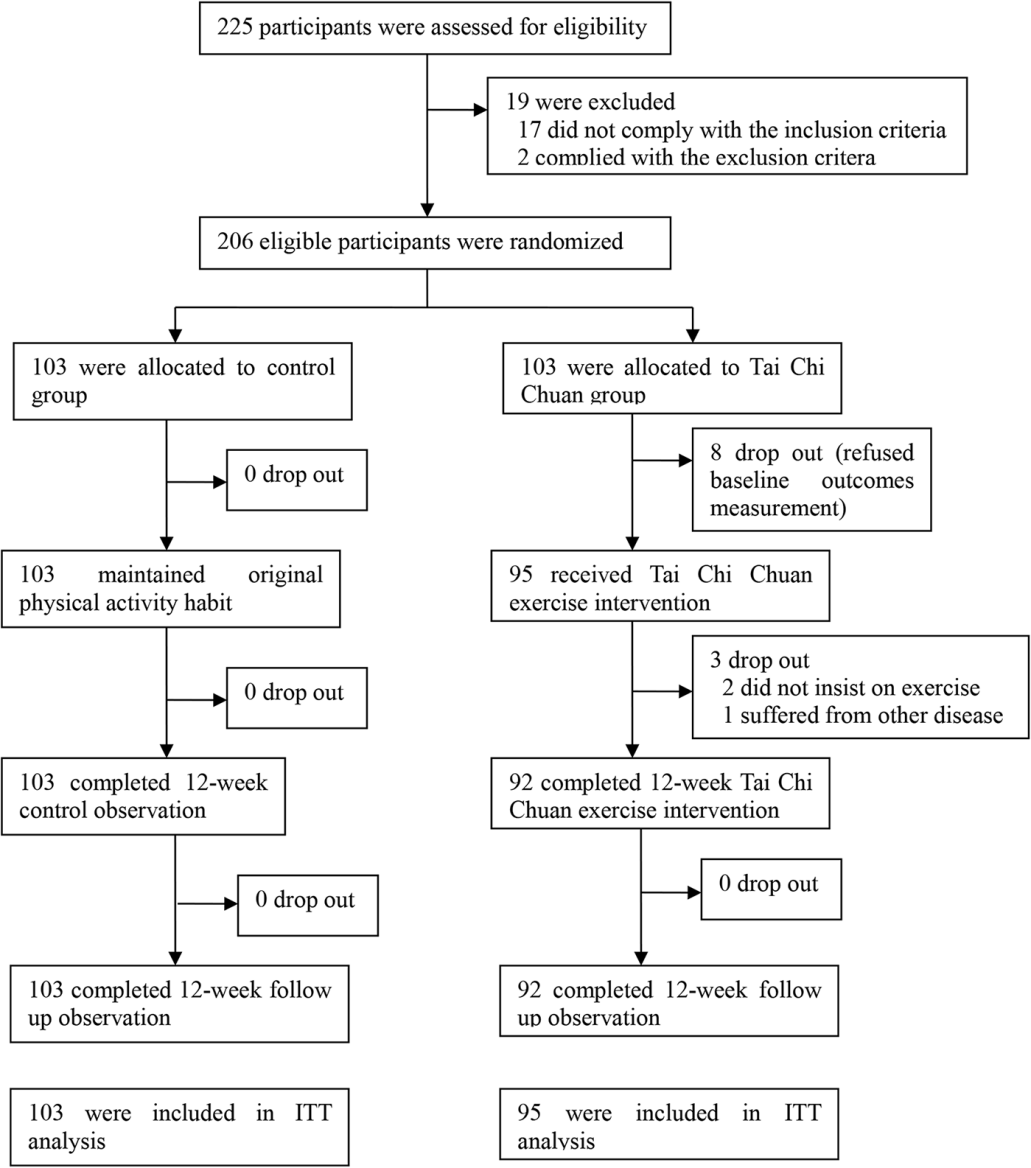
Participant flow through the study reported in a CONSORT diagram.

Fig 1 illustrates participant flow as recommended by the CONSORT statement. Eight participants in the TCC group dropped out before baseline characteristic measurement and, therefore, were excluded from the ITT analysis. During the entire study period, consisting of the 12-week intervention period and the 12-week follow-up period, only three participants in the TCC group dropped out and no participants dropped out of the control group. Therefore, 92 (96.8%) participants in the TCC group and 103 (100%) participants in the control group completed the entire study. The Fisher’s exact test indicated no significant difference in dropout rate between the two groups (*P* = 0.109).

Of the 198 eligible participants, the average ages were 20.6 years and the majority of them (67.2%) were female. Self-reported average exercise time was approximately 33.78 minutes per day in the previous month. There was no significant difference between groups for baseline demographic characteristics, including sex proportion, average age, self-reported average exercise time, height, and weight (Table 1). Table 2 shows the comparison of participant daily physical activity and daily sedentary time between groups during the study period. The average durations of high intensity activity, low intensity activity, and sedentary activity were not significantly different between the two groups, both for the 12-week intervention period and for the 12-week follow-up period. The average time performing moderate intensity activity in the TCC exercise group was higher than that for the control group for the 12-week intervention period (*P* = 0.001), but not for the 12-week follow-up period.

**Table 1:** Baseline characteristics between the TCC exercise and control groups.

Characteristics	TCC group (n = 95)	Control group (n = 103)	Total
Sex: Females (%)	62 (65.3)	71 (68.9)	133 (67.2)
Age–yr (Mean±SD)	20.7±1.1	20.6±1.2	20.6±1.1
Height-m (Mean±SD)	162.3±7.4	162.4±7.9	162.4±7.6
Weight-kg (Mean±SD)	54.9±8.7	54.5±8.1	54.7±8.4
Baseline physical activities time*–min	35.35±20.89	32.34±18.71	33.78±19.80

**Table 2:** Comparison of average activities time during the 12-week intervention and 12-week follow-up period between groups (hours).

Characteristics	TCC group (n = 95)	Control group (n = 103)	*P* value
Average activities time during the intervention period (1–12 week)*			
Sedentary time	8.46±2.04	8.72±2.86	0.204
Low intensity activities time	1.85±0.90	1.73±0.92	0.237^#^
Moderate intensity activities time	1.06±0.33	0.56±0.37	0.000
High intensity activities time	0.17±0.22	0.21±0.32	0.724
Average activities time during the follow-up period (13–24 week) *			
Sedentary time	7.48±2.64	7.64±3.21	0.311
Low intensity activities time	2.22±1.33	2.16±1.39	0.398
Moderate intensity activities time	0.85±0.93	0.84±0.73	0.689
High intensity activities time	0.22±0.48	0.23±0.43	0.591

*Sedentary times were defined as the time of sitting down with waking state, such as attending classes, reading, playing computer, etc. *Low intensity activities* were defined as those with energy consumption less than 3.0. Metabolic Equivalents (METs) including walking, general housework, mild stretching exercise, etc. *Moderate intensity activities* were defined as those with energy consumption range from 3.0 to 6.0 METs including TCC, brisk walking, jogging, cycling, stretching exercise, etc. *High intensity activities* were defined as those with energy consumption over 6.0 METs including running, playing ball games, mountaineering, etc.

# Mann-Whitney U test.

### Adherence to the Trial

Of the 95 participants in the TCC exercise group, 92 (96.8%) completed the 12-week TCC training. However, due to conflicts between training time and other commitments, such as class meetings, course examinations, and so on, not all individuals in the TCC group adhered to the complete training plan (that is, one hour per session, five sessions per week for 12 weeks). 19 participants had an attendance rate (actual training days/planned training days) of 75% or less, 42 participants had a 76%-85% attendance rate, and 34 participants attained more than an 85% attendance rate.

### The Effect of Tai Chi Chuan Exercise on Primary Physical and Psychological Outcomes

Table 3 shows the changes in primary outcomes for the two groups from baseline to the end of the 12-week intervention period, and to the end of the 12-week follow-up period. Of the four parameters for balance ability, the open eyes perimeter and the closed eyes perimeter showed significant improvement after the 12-week exercise program for participants in the TCC exercise group. For the TCC group, the open eyes perimeter and closed eyes perimeter decreased a median of 21 and 33, respectively, and the control group had a median decline of 7 and 16, respectively, representing a significant difference after adjustment for baseline using a general linear model (*P* = 0.0414, *P* = 0.008, respectively). Furthermore, the significant difference between the comparison groups for the closed eyes perimeter was still present at end of the 12-week follow-up period, with a median decrease of 58 in the TCC exercise group compared with a median decrease of 33 in the control group (*P* = 0.003). Although the changes in the other two parameters for balance ability, open eyes ellipse area and closed eyes ellipse area, had a greater decrease in the TCC exercise group than in the control group both at 12 weeks post-intervention and at the end of the 12-week follow-up period, a significant difference was not observed. Participant flexibility showed a clear improvement after the 12-week intervention for the TCC group. The length of flexibility increased a mean of 1.4-centimeters in the TCC group compared with only a mean of 0.7-centimeters in the control group, which was a significant difference after adjustment for its baseline (*P* = 0.039). For two parameters of lower limb proprioception function (right lower limb ATE and left lower limb ATE), no significant changes were observed between the TCC exercise group and the control group post-intervention or at the end of follow-up. Similarly, the primary psychological outcomes of well-being, including self-efficacy, self-reported psychological symptoms, stress and attention, were not significantly different between the TCC group and the control group post-intervention or at the end of the follow-up period.

**Table 3:** Primary outcomes on physical and psychological health (Mean±SD/ Median (Inter-quartile range).

Outcomes	Groups	Numbers of participants	Baseline	*P* value	End of 12 week intervention period	Difference of 12 week intervention to baseline	*P* value/ *P* value**^§^**	End of 12 week follow-up period	Difference of 12 week follow-up to baseline	*P* value/ *P* value**^§^**
**Balance ability**										
Open eyes Perimeter	TCC	95	263.6(208.4~343.6)		235.6(191.8~314)	-21(-66~24)	0.1696/	257.2(219.2~314)	-9(-72~44)	0.419/
	Con	103	250(217~306)	0.822	261(216~300)	-7(-59~48)	**0.0414^§^**	254(212~304)	1(-64~43)	0.356**^§^**
Open eyes Ellipse Area	TCC	95	155(106.4~273.8)		141(89.6~227.6)	-15(-78~29)	0.839/	141.6(104~207)	-14(-90~28)	0.523/
	Con	103	153(109~254)	0.819	146(99~215)	-10(-85~39)	0.746**^§^**	145.8(103.6~218)	-4(-95~46)	0.294**^§^**
Close eyes Perimeter	TCC	95	433.2(323~535)		370.5(284~454)	-33(-108~25)	0.719/	384.2(291~443)	-58(-139~25)	0.729/
	Con	103	392.2(300~554)	0.536	367(293~483)	-16(-137~50)	**0.008^§^**	360(281.8~471)	-33(-131~45)	**0.003^§^**
Close eyes Ellipse Area	TCC	95	334.2(213~677)		294(175~460)	-57(-243~47)	0.501/	268.6(181~405)	-89(-268~32)	0.420/
	Con	103	348.4(201~663)	0.741	300(188~496)	-44(-272~102)	0.983**^§^**	294(198~436)	-34(-289~93)	0.114**^§^**
**Lower limb proprioception function**										
Right lower limb ATE (%)	TCC	95	27(22~34)		20(16~23.2)	-8(-13~-2)	0.768/	20(17.8~24)	-7(-14~-2)	0.435/
	Con	103	27(20~34)	0.742	19(17~24)	-6(-13~-1)	0.554**^§^**	21(18~24)	-5(-13~0)	0.812**^§^**
Left lower limb ATE (%)	TCC	95	28(22~33)		21(17.4~24.7)	-8(-13~1)	0.706/	22.6(19.2~27.6)	-4(-11~1)	0.458/
	Con	103	28(22~34)	0.936	21(18~25)	-6(-13~-1)	0.744**^§^**	24(20~28)	-3(-11~1)	0.728**^§^**
**Flexibility**, (centimeter, cm)	TCC	95	12.21±7.67		14.09±7.40	1.4(0~3.6)	**0.021/**	10.69±7.79	-1.6(-3.5~1.1)	0.184/
	Con	103	12.16±6.45	0.963	12.88±6.57	0.7(-1.1~2.9)	**0.039** **^§^**	9.81±7.53	-2.3(-4.7~0.3)	0.296**^§^**
**Psychological outcomes**										
Self-efficacy, score	TCC	95	2.56±0.43		2.59±0.44	0(-0.2~0.2)	0.463/	2.59±0.48	0.04±0.43	0.480/
(GSES score)	Con	103	2.47±0.39	0.141	2.54±0.45	0(-0.2~0.3)	0.866**^§^**	2.55±0.47	0.1(-0.2~0.2)	0.580**^§^**
Psychological symptom, score	TCC	95	139(118~159)		136.42±31.85	-7(-20~6)	0.476/	123.5(107~152.8)	-12(-25~1)	0.280/
(SCL-90 scale, score)	Con	103	145(126~168)	0.136	139.78±32.51	-10(-23~4)	0.392**^§^**	127(111~156)	-12(-24~4)	0.688**^§^**
Stress, score (CPSS scale)	TCC	95	23.48±5.35		22.99±5.65	-0.49±5.19	**0.075/**	22.55±6.44	-1.01±5.45	0.336/
	Con	103	24.93±5.29	0.056	24.51±6.36	-0.42±4.72	0.454**^§^**	23.47±6.65	-1.46±5.23	0.417**^§^**
Attention, second	TCC	95	201(176~241)		196.5(169.3~232.8)	-12(-41~15)	0.560/	190(155~236)	-14(-44~20)	0.569/
(Schulte Grid test)	Con	103	212.79±46.32	0.692	195(165~236)	-10(-51~29)	0.652**^§^**	202(159~232)	-17(-47~18)	0.788**^§^**

*P* value; *t*-test for normal distribution or Mann-Whitney U test for non-normal distribution.

^**§**^ *P* value: adjusted for baseline value of open eyes perimeter, open eyes ellipse area, close eyes perimeter, close eyes ellipse area, right lower limb ATE, left lower limb ATE, flexibility, self-efficacy, psychological symptom, stress, or attention, respectively by using general linear model.

### The Effect of Tai Chi Chuan Exercise on Secondary Physical and Psychological Outcomes

Compared with controls, no significant effect of TCC exercise on cardio-pulmonary function, blood pressure, resting heart rate, self-esteem, mood and mindfulness, quality of life, or quality of sleep could be demonstrated post-intervention or at the end of the follow-up period, even after adjusting for baseline. Table 4 lists the analysis results for secondary outcomes between the two groups (Table 4).

**Table 4:** Secondary outcomes on physical and psychological health(Mean±SD/ Median (Inter-quartile range).

Outcomes	Groups	Numbers of participants	Baseline	*P* value	End of 12 week intervention period	Difference of 12 week intervention to baseline	*P* value/ *P* value**^§^**	End of 12 week follow-up period	Difference of 12 week follow-up to baseline	*P* value/ *P* value**^§^**
**Cardio-pulmonary function**										
Step testing	TCC	95	53.75±14.72		47.59±7.43	-6.16±16.33	0.630/	49.46±8.63	-4.28±15.85	0.672/
	Con	103	54.41±15.18	0.756	48.14±8.40	-6.27±16.48	0.655**^§^**	48.97±7.68	-5.44±16.14	0.586**^§^**
Vital capacity, ml	TCC	95	2815±787		2665±792	-149±488	0.862	2817±751	1.85±508	0.608/
	Con	103	2832±781	0.879	2684±740	-148±373	0.531**^§^**	2872±766	40±472	0.835**^§^**
SBP, mmHg	TCC	95	116.89±13.97		111.25±11.65	-5.7±14	0.817/	112.35±12.74	-4.12±13.57	0.655/
	Con	103	115.94±11.41	0.598	111.64±12.32	-4.30±10	0.852**^§^**	113.19±13.72	-2.75±10.83	0.588**^§^**
DBP, mmHg	TCC	95	68.92±9.01		65.86±8.93	-3.3±10.4	0.896/	65.67±8.84	-3.1±8.8	0.557/
	Con	103	68.9±10.0	0.992	65.71±7.76	-3.2±10.6	0.662**^§^**	66.36±7.66	-2.5±10.2	0.323**^§^**
Rest heart rate	TCC	95	82.26±12.97		83.13±13.87	0.86±12.33	0.863/	85.12±15.03	2.58±16.02	0.6464
	Con	103	80.84±11.45	0.414	83.46±12.78	2.61±12.25	0.359**^§^**	83.64±13.31	2.80±14.52	0.818**^§^**
**Psychological outcomes**										
Self-esteem, score (SES scale)	TCC	95	31.58±3.79		30(29~34)	0.14±2.56	0.132/	30(29~33)	-0.47±3.34	0.460/
	Con	103	30.64±3.43	**0.069**	30(29~33)	0.11±3.00	0.902**^§^**	30(29~32)	0.06±3.68	0.455**^§^**
Mood and mindfulness, score	TCC	95	100.43±17.43		105(95~120)	6.57±18.91	0.454/	102.4±19.34	1.98±18.51	0.584/
(POMS scale)	Con	103	105.86±17.03	**0.028**	108(95~121)	3.18±17.32	0.351**^§^**	104.56±18.54	-1.31±17.59	0.255**^§^**
Quality of life, score	TCC	95	56.06±6.10		54(51~59)	-0.77±6.35	0.445/	55.25±7.23	-0.81±5.34	0.944/
(WHOQOL-BREF scale)	Con	103	54.67±6.41	0.119	54(50~59)	-0.31±5.43	0.636**^§^**	55.41±7.62	0.73±5.65	0.663**^§^**
Quality of sleep, score	TCC	95	3.77±2.15		4.36±1.81	0.58±2.11	0.425/	4.11±2.09	0.34±2.18	0.615/
(PSQI scale)	Con	103	3.84±1.78	0.813	4.54±2.10	0.71±2.14	0.768**^§^**	4.27±2.05	0.44±2.13	0.887**^§^**

*P* value: *t*-test for normal distribution or Mann-Whitney U test for non-normal distribution.

^**§**^ *P* value: adjusted for baseline value of step testing, vital capacity, SBP, DBP, rest heart rate, self-esteem, mood and mindfulness, quality of life, or quality of sleep, respectively by using general linear model.

### Safety

No adverse events (AEs) related to the TTC exercise occurred during this trial.

## Discussion

In this randomized controlled trial comparing TCC exercise with usual exercise controls on the physical and mental health of college students, we found significant improvements with regular TCC exercise for two parameters of balance ability (open eyes perimeter and closed eyes perimeter) and for flexibility over a 12-week intervention period. Furthermore, a significant benefit for closed eyes perimeter was observed over the additional 12-week follow-up period, indicating a possible long-term benefit to practicing TCC exercise. No adverse events related to TCC exercise were reported during the intervention period, suggesting that TCC exercise is safe.

TCC is a traditional Chinese mind-body exercise. Its unique characteristics include mind concentration with breathing control, whole-body exercise in a semi-squat posture, and continuous, curved, and spiral body movement. When TCC is being practiced, deep diaphragmatic breathing with mind concentration is integrated into body motions to achieve a harmonious balance between body and mind. Therefore, TCC is beneficial to the physical, emotional, and social function of the practitioner [23]. Previous studies have demonstrated that regular TCC exercise has significant benefits on health promotion and that TCC may promote aerobic capacity, muscular strength, and balance capability [22–24]. The effectiveness of TCC on improving psychological symptoms, such as anxiety, depression, and stress, has also been reported [29–30, 45–46]. However, most of these studies focused mainly on the health effect of TCC for patients with various chronic diseases or for an older aged population. Several studies have investigated the association between TCC and the physical or mental health of college students or young adults [47–49]. A study conducted with a before and after design reported that Tai Chi exercise of one hour sessions, twice per week for three months improved the physical function, general health, and mental health, including social function, emotional function and vitality, of college students [47]. Another quasi-experimental study with repeated measures using TCC training of 50 minute sessions, 2 times per week for 15 weeks also suggested that TCC was beneficial for sleep quality, mood, and perceived stress of college students aged 18–48 years [48]. However, the conclusions could not be confirmed due to design limitations in these studies. In the present study, we scrupulously designed a randomized, parallel controlled, assessor- and statistician-blinded trial to evaluate the effectiveness and safety of TCC for college students. Our results indicated that regular TCC exercise could significantly improve flexibility and balance ability in college students compared with usual exercise. However, no significant improvement in lower limb proprioceptive function, cardio-pulmonary function, or psychological outcomes, including self-reported psychological symptoms, stress, self-esteem, mood and mindfulness, and quality of life and sleep, was found for TCC exercise. One possible explanation for this result is that all participants enrolled in this trial were young (aged 18–23 years old) and apparently healthy college students, in contrast to those with sedentary habits or psychological troubles. Second, although those with long-term regular exercise were excluded, including members of the Wushu association, Taekwondo association, and Dance association, the duration and type of usual exercise of the control group was not limited. Therefore, subjects in both groups may have been involved in some regular sporting exercise, such as jogging, football, basketball, badminton and ping-pong, that could potentially affect the difference between the comparison groups. Third, it is possible that the 12-week intervention period for TCC exercise is not sufficient to identify significant differences for the young and apparently healthy college student population. In addition, the study sample size of 206 participants may not provide adequate power to identify the effect of TCC exercise on the physical and psychological outcomes in the college student population.

We recognize several inherent limitations in our study. First, ideally everyone involved in a RCT should be blinded as an important safeguard against bias, particularly when assessing subjective outcomes, but blinding of participants and healthcare is often difficult, if not impossible, in non-pharmacological trials [50]. In our trial, it was impossible to blind the TCC instructors and college students. Nevertheless, we kept the randomized allocation sequence and the blind codes of allocation with an independent project manager who did not take part in any part of the research. The blind codes were not disclosed until the data analysis was completed. All physical outcomes such as balance ability and lower limb proprioception were measured by blinded operators from the Evaluation Department of Rehabilitation Hospital (Affiliated to FJTCM) or from the faculties at the Physical Exercise Department of FJTCM. Second, the fact that all eligible participants came from the same university may potentially limit the generalizability of the results. Third, individual adherence to the TCC exercise regimen was not complete because training time conflicted with other commitments, thus potentially underestimating the effect of the TCC intervention. Nonetheless, excellent protocol adherence, strict performance supervision, and no significant loss to follow-up or withdrawal were helpful in strengthening the findings.

In conclusion, this study demonstrates that regular TCC exercise can promote improvement in flexibility and balance capability to an extent in a college student population. Further studies with larger sample sizes and longer intervention periods are necessary to confirm these conclusions.

## Supporting Information

S1 File CONSORT checklist. (DOC)

S2 File Ethic (English version). (DOC)

S3 File Funding (English version). (DOC)

S4 File Study protocol (English version). (DOC)

S5 File Study protocol (Chinese version). (DOC)
