# Supplementary material for: Effectiveness of Tai Chi on Physical and Psychological Health of College Students: Results of a Randomized Controlled Trial
Source: PLoS One. 2015 Jul 6;10(7):e0132605. doi: 10.1371/journal.pone.0132605 (PMC4492604; doi:10.1371/journal.pone.0132605)
Supplement: S2 File — (DOC) [file pone.0132605.s002.doc]

**The Comment on the Ethical Review of the Study--** ***The effectiveness of Tai Chi on physical and*** ***psychological health of college students: study protocol for a randomized controlled trial***

The objective of the study-- ***The effectiveness of Tai Chi on physical and psychological health of college students: study protocol for a randomized controlled trial***, conducted by Guohua Zheng, Xiulu Lan, et al., is to evaluate the effect of Tai Chi Chuan for physical and psychological health of college students.

The study design of the protocol does not use any identifiable specimens, and the researchers strictly follow the *Declaration of Helsinki*. Reviewed by the Ethics Committee, this project does not go against with the ethical principles, and we accepted the application of the implementation of the study on the premise of informed consent.

The Ethics Committee of Fujian University of TCM

April 12th, 2013
