# Supplementary material for: Effectiveness of Tai Chi on Physical and Psychological Health of College Students: Results of a Randomized Controlled Trial
Source: PLoS One. 2015 Jul 6;10(7):e0132605. doi: 10.1371/journal.pone.0132605 (PMC4492604; doi:10.1371/journal.pone.0132605)
Supplement: S3 File — (DOC) [file pone.0132605.s003.doc]

State Administration of Traditional Chinese Medicine of the People's Republic of China

Science and Technology Division of SATCM (Dispatch No.[2013]11)

**Notice of the Project Approval of Special Scientific Research of Traditional Chinese Medicine of the Year 2013**

…………………………….

Project name (number): The community application of traditional Chinese medicine rehabilitation programme of dysfunction related to stroke and knee osteoarthritis (number: )

Project undertaking institution: Fujian University of Traditional Chinese Medicine

Project leader(s): Youxin Su, Jing Tao

Project completion time: from March 2013 to March 2016

Project total funding: 6.43 million

Office of SATCM

March 21, 2013
