# Supplementary material for: Effectiveness of Tai Chi on Physical and Psychological Health of College Students: Results of a Randomized Controlled Trial
Source: PLoS One. 2015 Jul 6;10(7):e0132605. doi: 10.1371/journal.pone.0132605 (PMC4492604; doi:10.1371/journal.pone.0132605)
Supplement: S4 File — (DOC) [file pone.0132605.s004.doc]

The effectiveness of Tai Chi on physical and psychological well-being of college students: study protocol for a randomized controlled trial

**Objective:**

We design a strict randomized controlled trail to systematically evaluate the effectiveness of Tai Chi Chuan (TCC) exercise for physical and mental health of college students. We expect to approach the objective and effective evidence to promote the universality of TCC.

**Methods/Design**

**Study design**

The study about the effectiveness of TCC on physical health and psychosocial well-being of college students is designed as a randomized, parallel controlled, assessor and statistician single-blinded trial. The allocation of participants will be equal (1:1) to the TCC exercise group (intervention group) and the control group. All assessment will be conducted by the blinded assessors at Fujian University of Traditional Chinese Medicine Affiliated Rehabilitation Hospital, and the gymnasium of FJTCM. The TCC exercise will be conducted at the gymnasium of FuJian University of TCM, and instructed by two qualified coaches. The entire trial program is illustrated in Figure 1.

**Sample size estimation**

Sample size estimation in this RCT is based on the expected improvement of the balance ability in 12-week TCC exercise in college students. The data of our preliminary experiment showed that the means and standard deviation of the balance ability (presented by motion of ellipse area which tested by standing with eyes closed on the flat of Pro-kin system) in the college students was 531.25 and 173.78. 15% improvement of balance ability will be expected after 12 week intervention. According to the same size of the estimation formula $\begin{matrix} n_{1}=n_{2}=2\left(\frac{z_{\alpha/2}+z_{\beta}}{M_{e}-M_{c}}\right)^{2}\delta^{2} \\ \end{matrix}$  (set α= 0.05, β= 0.10). It is estimated that a sample size of 93 participants per group will be required, considering 10% drop-out and exit. Therefore, we will recruit approximately a total of 206 participants, 103 participants in each group.

**Participants and Recruitment**

A total of 206 participants will be recruited at FJTCM. We plan to well-advertised the recruitment program through advertisements in the campus bulletin board and the campus radio. The interested students will contact the research assistants and will be screened according to the inclusion and exclusion criteria. The potential participant will be required to sign the informed consent if they fulfill inclusion criteria and do not have any exclusion criteria before enroll in this trial.

**Inclusion criteria**

Participants have to fulfill the following criteria:

①Age from 16 to 25 years.

②A full time freshman or [sophomore](app:ds:sophomore).

③Written informed consent for participation in the trial.

**Exclusion criteria**

Students would be ineligible if they meet any condition as following:

①A long-term exercising TCC or other Tai Chi derived movements

②A member of Students' Wushu Association or Taekwondo Association or Aerobic Association or Dance Association.

③Severe cardiovascular disease or Musculoskeletal Disease

**Withdrawal criteria** Participants will be withdrawn from the trial if they present following conditions:

①Poor compliance (mean compliance < 85% at the last estimation) or noncompliance.

②Occurrence of a serious adverse event.

③Initiative exit.

④Unable to progress because of sudden disease.

⑤Members in the control group have been regularly engaged in the TCC exercise.

**Ethical consideration**

This study protocol adheres to the principles of the Declaration of Helsinki and has been approved by the ethics committee of FJTCM (No. 042). The written consent will be obtained form each participants before the baseline assessment. All participants will have the right to withdraw from the study at any time.

**Randomization and allocation concealment**

Participants will be randomly allocated to the TCC training group or the control group. Randomization will be performed at post-baseline assessment. The randomization list will be generated via Statistical Analysis System (SAS, version 9.1) by an independent non-investigator who works in Center for EBM of Academy of Integrative Medicine FuJian China. The allocation sequence will be concealed through a password access files which will be taken care of by the project manager. The eligible participants will be informed the allocated result by the project manager after post-baseline assessment.

**Blinding**

It is impossible to blind the participants and TCC coaches in this trial. Nevertheless, we will assign a specified project manager to be in charge of the random allocated sequence and blind code of allocation in which TCC training group or control group will be replaced by the alphabet A or B. In addition, we will define each investigators a well-defined obligation; the project manager and TCC coaches will be not take part in the assessment of outcome; the outcome assessors and the statistic analyzer will be not involve in the participants’ recruitment and allocating. The allocation sequence and blind codes will be preserved hold by an independent project manager until the statistic analysis is completed.

**Intervention**

**TCC training group**

Participants allocated to the TCC training groups will receive 12 weeks of TCC training. The TCC training will be instructed by two experienced TCC coaches, who have been qualified and engaged in the teaching of TCC course for at least 15 years. The 24 forms simplified TCC, which is recommended as the popularity health sport by General Administration of Sport of China, will be applied to members of the intervention group. Participants of TCC training group will be gathered to exercise at the campus gymnasium together. TCC training will be performed for 60 minutes per time and also include 10 minutes of warm-up (e.g., weight shifting, arm swinging, gentle stretches of the neck) at a frequency of five days a week. Participants will be taught the “meditation through movement” art of TCC, which includes the rationale states of TCC constitute, action essence of TCC, the TCM philosophy opinion of TCC, and deep breathing method. The instruction of TCC will be both verbal and visual. The systematic approach has been designed by two coaches and can be viewed on DVD.

**Control group**

No specific exercise will be administered on the participants in control group. They will be informed to maintain their original lifestyle.

**Intervention regimen**

The intervention period in this trial will last 12 weeks. All participants will be required to record physical activity diaries including the type and intensity of physical activity or exercises, as well as the sedentary time, sleeping time everyday throughout this study.

**Follow-up period**

During the 12-week follow-up period, all of the participants will return to their original lifestyles, but be required to record their daily physical activities or sport information. The primary outcomes will be re-measured at end of follow-up period.

**Outcomes measurement**

The primary or secondary outcomes will be tested at baseline, 12 and 24 week post-intervention. A schedule of assessments that will be carried out is listed in Table 1. The balance ability and [lower](app:ds:lower) [limbs](app:ds:limbs) proprioception will be assessed at Rehabilitation Hospital Affiliated to FJTCM by three experienced [rehabilitation](app:ds:rehabilitation) [therapist](app:ds:therapist)s who do not involve in this study. The physical fitness test, [cardio-pulmonary](app:ds:cardio-pulmonary) [function](app:ds:function), flexibility, vital capacity, and weight will be conducted at campus gymnasium by the physical education teachers. The outcome assessors of this trial will be in charge of the assessment of relevant psychology scales including self-efficiency, attention, stress self-esteem, and quality of sleep.

**Primary outcome measures**

1. Balance ability

It will be tested by standing with eyes open for 30 seconds and standing with eyes closed for 30 seconds via Pro-kin system(Machine type: produce by *Italy Tecnobody. S.R.L company, model PK254P*)

2. Lower [limbs](app:ds:limbs) proprioception: both left and right legs will be tested via Pro-kin system (*Machine type: produce by Italy Tecnobody. S.R.L company, model PK254P*).

3. Flexibility: will be tested with Sit and Reach flexibility test equipment (Machine type: *Beijing ZhongTi Tongfang Co., Ltd., model CSTF-TQ-5000*).

4. Self-efficiency: *The* *Self-Regulatory Self-Efficacy Scale* will be used to test self-efficiency.

5. Psychological symptom score: will be assessed by using *SCL-90 scale* .

6. Attention span: will be assessed with *Schulte grid (8*8) scale.*
7. Stress: will be measured with *Chinese Perceived Stress Scale* .

**Secondary outcomes measures**

1. [cardio-pulmonary](app:ds:cardio-pulmonary) [function](app:ds:function): will be evaluated indirectly by step test, vital capacity, blood pressure and rest heart rate. Step testing：step test will be conducted by Electronic step test instrument（stairs with 30 [centimeter](app:ds:centimeter)s of step height will be used for males, and 25 [centimeter](app:ds:centimeter)s of step height will be used to females）(Machine type: *Beijing ZhongTi Tongfang Co., Ltd., model CSTF-TZ-5000*). Vital Capacity: will be tested by electronic vital capacity instrument (Machine type: *Beijing ZhongTi Tongfang Co., Ltd., model CSTF-FH-5000*); blood pressure and rest heart rate will be tested by electric sphygmomanometers produced by the *Omron Corporation, China* (product type: *HEM-746C*).

2. Self-esteem: will be assessed with self-esteem scale.

3. Mood and mindfulness: will be measured with *Profile of Mood States (POMS)*.
4. Quality of life: will be tested with *WHOQOL-BREF scale*.

5. Sleep quality: will be measured by using the *Pittsburgh Sleep Quality Index (PSQI)*.

**Safety** **evaluation**

Any adverse events (defined as any functional lesion caused by the intervention, such as knee joint or ankle sprain, knee soreness, lumbar muscle strain and so on) will be recorded on case report form (CRF) during intervention period. If any adverse event occurs, the coaches or project managers will provide the corresponding treatment to the participant. The adverse events will be immediately reported to the primary investigator and ethics committee to decide if the participant needs to withdraw the trial.

**Statistical analyses plan**

SPSS21.0 statistical analysis software will be used to analyze data. The primary outcomes of participants who are randomized and received at least one treatment week will be carried by the intention-to-treat (ITT) analysis. Per-protocol subjects analysis（PPS）of the primary outcomes will include participants who have completed the 24-week study without major protocol violations and have compliance rate > 85%. We will compare the results of the ITT with that PP analysis to check-up whether the results are consistent or not. The continuous variables will be expressed using means with standard deviations or medians with ranges. For the variables with a normal distribution, statistical comparisons between the groups will be made by using a *t* test. If the variables have a non-normal distribution of ordinal level, statistical comparison between groups will be made using the Mann-Whitney *U* test. Measures with a discrete distribution will be expressed as percentages and analyzed by theχ^2^ or Fisher’s exact test as appropriate. A general linear model or Logistic regression model will be applied to adjust the confounding influence if necessary. The *p* value is less than or equal to 0.05 will be considered as a test by the difference has statistical significance.

Participants’ recruitment

Eligible patients n=206

Baseline assessment

Exclusion (reasons): n=

Not meeting inclusion criteria: n=

Declined to participate: n=

Other reasons: n=

Randomization allocation

TCC training group

n=103

Control group

n=103

Drop out (reasons): n=

TCC training group: n=

Control group: n=

Outcome assessment after intervention: balance ability, lower limbs proprioception, flexibility, physical fitness, self-efficiency, psychological symptoms ,attention, stress, self-esteem, mood and mindfulness, quality of life, quality of sleep, safety outcomes.

Follow up for 12 weeks

Lost to follow up: n=

TCC training group: n=

Control group: n=

Outcome assessment after follow-up period

Included analysis: n=

TCC training group: n=

Control group: n=

Statistic analysis

Excluded from analysis: n=

TCC training group: n=

Control group: n=

**Recruitment**

**Allocation and intervention**

**Follow up**

**Analysis**

**Figure 1 Flow diagram of participants**

| **Table 1 Trial Processes Chart**   | Items | Before enrollment (week) | TCC training phase(week) | Training end (week) | Follow-up (week) | Follow-up end(week) | | --- | --- | --- | --- | --- | --- | | **-2-(-1)** | **1-12** | **13** | **13-24** | **25** | | Inclusion criteria | * |  |  |  |  | | Exclusion criteria | * |  |  |  |  | | Informed consent | * |  |  |  |  | | Baseline | * |  |  |  |  | | Randomization | * |  |  |  |  | | Self-esteem | * |  | * |  | * | | Mood and mindfulness | * |  | * |  | * | | Self-efficiency | * |  | * |  | * | | Psychological symptom score | * |  | * |  | * | | Stress test | * |  | * |  | * | | Flexibility | * |  | * |  | * | | Step testing | * |  | * |  | * | | Rest heart rate | * |  | * |  | * | | Vital Capacity | * |  | * |  | * | | Systolic [blood](app:ds:blood) [pressure](app:ds:pressure) | * |  | * |  | * | | [Diastolic](app:ds:diastolic) [blood](app:ds:blood) [pressure](app:ds:pressure) | * |  | * |  | * | | Balance | * |  | * |  | * | | [Lower](app:ds:lower) [limbs](app:ds:limbs) proprioception | * |  | * |  | * | | Attention span | * |  | * |  | * | | Quality of life | * |  | * |  | * | | Quality of sleep | * |  | * |  | * | | Safety outcomes |  | * |  | * |  | | Self-report diaries |  | * |  | * |  | |
| --- | --- | --- | --- | --- | --- | --- | --- | --- | --- | --- | --- | --- | --- | --- | --- | --- | --- | --- | --- | --- | --- | --- | --- | --- | --- | --- | --- | --- | --- | --- | --- | --- | --- | --- | --- | --- | --- | --- | --- | --- | --- | --- | --- | --- | --- | --- | --- | --- | --- | --- | --- | --- | --- | --- | --- | --- | --- | --- | --- | --- | --- | --- | --- | --- | --- | --- | --- | --- | --- | --- | --- | --- | --- | --- | --- | --- | --- | --- | --- | --- | --- | --- | --- | --- | --- | --- | --- | --- | --- | --- | --- | --- | --- | --- | --- | --- | --- | --- | --- | --- | --- | --- | --- | --- | --- | --- | --- | --- | --- | --- | --- | --- | --- | --- | --- | --- | --- | --- | --- | --- | --- | --- | --- | --- | --- | --- | --- | --- | --- | --- | --- | --- | --- | --- | --- | --- | --- | --- | --- | --- | --- | --- | --- | --- | --- | --- | --- | --- | --- |
