# Supplementary material for: Effectiveness of Tai Chi on Physical and Psychological Health of College Students: Results of a Randomized Controlled Trial
Source: PLoS One. 2015 Jul 6;10(7):e0132605. doi: 10.1371/journal.pone.0132605 (PMC4492604; doi:10.1371/journal.pone.0132605)
Supplement: S5 File — (DOC) [file pone.0132605.s005.doc]

**太极拳运动对大学生身体心理健康影响效果的随机对照**

**研究计划书**

**一、**研究背景

大学生是祖国和社会的未来，肩负着建设祖国的重任，其身心健康是社会关注的焦点。随着生活水平的提高大学生的形态发育水平提高，身高体重等指标呈增长趋势，营养状况明显改善，但是也存在很多不容忽视的问题，比如耐力速度爆发力等呈下降趋势。肥胖、超重、视力不良等检出率依然严重[1,2]。影响现代大学生身体健康的主要因素有缺乏体育锻炼、生活无规律、缺乏充足的睡眠时间等。多项研究表明不合理的饮食结构，生活方式的现代化和运动安排的不合理均是导致当代大学生体质下降的主要原因[3-5]。大学生不仅要面对当今社会激烈的竞争，还要面对来自学业、人际、家庭、就业等各方面的压力。不仅要掌握现代化的科学技术，具有较全面的能力，而且要有强健的体魄、健全的人格、健康的心理素质。因此，寻找有效提高大学生身心素质的方法具有重要意义。

体育运动作为一种有效改善健康状况的手段，已受到人们的高度认可和欢迎。作为我国传统健身运动项目的太极拳，以其“刚柔相济、虚实分明、松柔缓慢、修心养性”等特点广受欢迎。长期坚持太极拳练习可能有效地改善练习者身体机能，改善心理状态，促进练习者身心健康得到统一、协调的发展[6-10]。大量研究显示体育锻炼和活动和身心练习能够降低冠心病、高血压、肥胖、糖尿病和骨质疏松症的发病率和死亡率，并能够提高普通人的心理状况 [10-15] 。

目前,太极拳对身心健康的影响的证据依然尚不充分，依然存在以下问题1.研究未采用严格的随机对照法；2.未对结果评价者及数据统计分析者采用盲法；3.多数研究样本含量较小。以上原因导致实验偏倚大，影响太极拳运动的运用及推广。我们拟采用随机对照研究，在大学生群体中开展规范化太极拳训练，通过检测练习者身体机能和心理因素的指标变化，系统分析太极拳运动对人体身心健康的影响，以期得到客观、科学的研究证据，为提高太极拳运动普及和推广提供科学依据。

**二、主要研究内容**

采用随机对照设计评价太极拳对大学生身体心理健康影响的效果。

**三、研究方案与技术路线**

**1.样本量计算**

以太极拳对平衡功能的改善作为主要效应指标对样本进行估算，预实验得出闭眼平衡的运动椭圆面积的均数$\overline{x}$=531.25，σ=173.7883。以闭眼平衡的运动椭圆面积提高15%为预期目标，α取0.05，β取0.1，根据公式：$\begin{matrix} n_{1}=n_{2}=2\left(\frac{z_{\alpha/2}+z_{\beta}}{M_{e}-M_{c}}\right)^{2}\delta^{2} \\ \end{matrix}$得出n_1_= n_2_=94，允许10%失访率，即得出实验组103人，对照组103人。

**2.研究对象招募与分组**

**2.1对象招募：**面向福建中医药大学一、二年级大学生招募合格的志愿者。

**2.2随机序列的产生、隐藏与对象****分组：**根据估算的样本含量大小，由学校循证医学中心采用统计软件SAS9.1程序产生随机分配序列，将合格的研究对象按纳入顺序分成空白对照组和太极拳训练组。随机分配序列由课题组指定的项目管理员保管，不给其他人员知晓。

2.3**设盲与揭盲：**本研究无法对研究对象设置盲法，采用单盲法对结局评价人员、数据管理和统计分析人员设置盲法。即用字母1、2代替CRF表中的组别通过SAS软件设置盲底并由项目管理员保存。待数据统计分析完成后，由项目管理员宣布组别的代码进行揭盲。

**3.纳入和排除标准**

**3.1纳入标准**

①年龄16-25岁；

②知情同意，志愿参加；

③大学一年级或二年级在读；

符合以上标准者，可纳入研究。

**3.2排除标准**

①有长期规律练习太极拳或其他太极系列运动者；

②武术协会、跆拳道协会、散打协会、健美操协会、舞蹈协会的成员；

③严重心血管疾病、肌肉骨骼系统疾病者。

符合以上任何一条标准者,不予纳入研究。

**4.剔除标准、中止标准及处理原则**

**4.1剔除标准**

①资料填写不完整导致主要结局指标信息缺失者；

②填写虚假信息或报告虚假结局者；

**4.2 中止标准**

①不能坚持训练者；

②不按训练要求进行练习者；

③试验期间出现突发疾病不能继续参加训练者；

④主动提出退出研究者；

⑤对照组成员在试验期间练习太极拳者；

⑥出现不良事件不宜继续练习者。

**4.3 剔除与中止的处理原则**

对于剔除、中止的受试者在其离开实验时进行问卷调查，明确记录原因及与试验的关系，并详细记录、评价结局指标，对严重不良事件者上报伦理委员会。

**5.干预方法**

**5.1**太极拳组: 采用1965年中国国家体委（后改组为国家体育总局）编排的24简化太极拳，由福建中医药大学具有教练资质的体育教师带领练习。包括起势、左右野马分鬃、白鹤亮翅、左右搂膝拗步、手挥琵琶、左右倒卷肱、左揽雀尾、右揽雀尾、单鞭、云手、单鞭、高探马、右蹬脚、双峰贯耳、转身左蹬脚、左下势独立、右下势独立、左右穿梭、海底针、闪通背、转身搬拦捶、如封似闭、十字手、收势24个动作。

**5.2对照组:**对该组被研究人员在自然状态下不采取任何干预措施，保持原有生活方式。

两组在干预过程中每天均须记录当天活动情况，归类分为静态作业、低等强度活动、中等强度活动、高等强度活动。同时记录当天所经历的生活事件，包括正性生活事件及负性生活事件。

**6.干预与随访**

**6.1干预时间：**试验组每次集中练习60分钟，每周练习5次，持续12周。每天练习时间为下午5点20分至6点20分。正式练习之前常规热身运动20分钟。

**6.2 随访**：以参与者自我报告的形式进行随访，随访12周，随访期间参与者按要求填写活动日志及相关情况表，随访结束时交到项目管理员。

**7.** **结局指标评价**

**7.1 主要结局指标**
①平衡功能：静态睁（闭眼）眼双足站立测试。机器型号：意大利 Tecnobody .S.r.l公司生产，型号PK254P。由国家中医药管理局康复重点研究室（福建省卫生厅重点科室—脊柱与关节运动功能康复研究室）的康复治疗师进行评定。
②下肢本体感。机器型号：意大利 Tecnobody .S.r.l公司生产，型号PK254P。由国家中医药管理局康复重点专科研究室（福建省卫生厅重点科室—脊柱与关节运动功能康复研究室）的康复治疗师进行评定。
③柔韧性(flexibility):坐位体前屈长度（L）：采用北京中体同方有限公司生产的坐位前屈测试仪（型号CSTF-TQ-5000），由福建中医药大学体育部进行测试。
④自我效能(self-efficiency)：采用一般自我效能量表测量(General Self-efficiency Scale,GSES）测量。
⑤症状评分：SCL90自评症状量表：90个条目，9个维度，即躯体强迫症状、人际关系敏感、抑郁、焦虑、敌对、恐怖、偏执和精神病性。
⑥注意力：舒尔特方格量表。
⑦压力：采用知觉压力量表（CPSS）测量。
**7.2次要指标**
①心肺功能:

台阶测试：根据《大学生体质健康标准》，采用北京中体同方有限公司生产的台阶试验测试仪（型号CSTF-TZ-5000），由福建中医药大学体育部进行测试。

肺活量：根据《大学生体质健康标准》，采用北京中体同方有限公司生产的肺活量测试仪（型号CSTF-FH-5000），由福建中医药大学体育部进行测试。

血压(收缩压、舒张压)、心率: 欧姆龙上臂式血压计（型号HEM-746c）。
②自尊(self-esteem)：采用自尊量表（SES）测量。
③情绪与情感（mood and mindfulness）：采用心境状态量表（POMS）测量。
④生活质量指数：采用生活质量量表（WHOQOL-BREF）测量。
⑤睡眠质量：匹茨堡睡眠质量指数（the pittsburgh sleep quality index,PSQI）

**7.3 安全性指标**

记录研究过程中出现副作用以及不良事件，并分析其原因，以各组不良事件发生率作为评价指标，不良事件发生率计算公式如下：

不良事件发生率%=（不良事件例数/该组总例数）×100%

**8.数据处理与分析**

**8.1数据管理：**研究报告表由研究对象、结果评价人员的填写，每个入选对象必须完成研究报告表。研究报告表由活动日志表、心理健康测量表、生活质量量表、心肺功能测量表、运动功能测量表、不良事件记录表等组成，完成的研究报告表由项目管理人员审核后，组织数据录入员进行数据录入并将原始表统一管理。

**8.2分析数据集的定义**

**①分析数据集（FAS）：**根据ITT原则对所有经随机化分组，并至少经历1周干预的全部对象进行分析。对其中因任何原因出组，未能观察到全部干预过程的研究对象资料，用最后一次观察数据结转为试验的最终结果。

**②符合方案数据集（PP）：**所有符合试验方案、依从性好、完成SRF规定填写内容的资料，对其干预效果进行统计分析。

**③安全性（Safety）人群：**所有经随机化分组，至少进行了一次安全性评估的研究对象，构成本研究的安全性人群。

**8.3统计分析计划**

统计分析将采用SPSS18.0统计分析软件进行计算。所有的统计检验均采用双侧检验，p值小于或等于0.05将被认为所检验的差别有统计意义。

**①基础值的均衡性分析：**比较两组人口学资料和其它基础值指标，以衡量两组均衡性；

**②有效性分析：**比较两组主、次要指标的差异以确定干预措施的有效性。

**③安全性分析：**比较两组不良事件发生率，并列表描述本次试验所发生的不良事件。

**8.4统计分析方法**

计量资料将采用均数±标准差或中位数（最小值，最大值）进行统计描述，t检验或秩和检验进行统计推断。计数资料采用频数（构成比）进行统计描述，x^2^检验精确概率法或非参数检验进行统计推断。当两组出现基线指标不均衡时，将应用一般线性模型或logistic回归模型进行校正性分析。

**9．质量监控**

**9.1志愿者知情同意的质量控制：**在知情同意书签署后，抽20%志愿者，询问志愿者对所参加的研究是否知情、对知情同意书所述内容是否知晓。

①知晓，则本步骤的质量监控获得通过。

②部分知晓或者完全不知晓，则要求执行知情同意的研究者进行宣教和沟通，然后抽取40%的志愿者再次询问，知晓则获得通过，部分知晓或完全不知晓则重复本步骤。

**9.2志愿者入组的质量监控：**严格按照纳入、排除标准筛选所有志愿者，是否符合入组标准。

①符合纳入标准且不符合排除标准者，则本步骤的质量控制获得通过。

②不合格者予以排除，记录人数及原因。

**9.3随机对照与盲法的质量监控：**项目管理员根据随机分组序列，按照筛选者的编号将合格对象随机分组，妥善保管盲底，待数据分析结束后统一揭盲。

**9.4干预过程的质量监控：**项目管理者监督教练员的指导训练过程，教练员应监督和指导干预组志愿者的训练过程。

**9.5统计分析的质量监控**

缺失值研究报告表中原则上不要能有缺失值，尤其是重要指标（主要的安全性指标）必须填写清楚。基本数据，如性别，出生日期、入组日期和各种观察等不得缺失，试验中测得的结果为零和未能测出者，均应有相应的符号表示，不能空缺，以便与缺失值区分。如果出现缺失值，应先查找原始资料，确定是否漏填，如确为漏填应补填；如原始资料中无记录，而该缺失值是涉及安全性的重要指标，则应通知受试者立即复查，以确保其安全；如缺失值影响结果判定，则该病例剔除。

**9.6数据管理质量控制：**项目管理者必须保存的文件清单，逐条核对。如有缺项，则要求相关人员补齐。

**【主要技术路线】**

**参考文献**

[1]孙晓静,张钦祥.当前大学生身体健康状况分析[J].中国科技信息,2005,(19):192-193.

[2]朱淑玲.在和谐社会中探析高校大学生身体健康状况[J].漯河职业技术学院学报,2010,(04):125-126.

[3] 王志敏.大学生生活节律与身体健康状况的调查研究[J].城市建设理论研究（电子版）,2011,(31).

[4]代建伍,刘丹,阮芳华.现代生活方式对大学生身体健康的影响及对策[J].科教导刊(上旬刊),2012,(03):106-107.

[5]齐新华.影响大学生身体健康的因素[J].黑龙江科技信息,2007,(15):176.

[6] 陈福刁,陈浩庆,张帼雄.太极拳锻炼对大学生身体机能的影响[J].沈阳体育学院学报,2006,25(4):121-123.

[7] 刘全,张勇.24式太极拳运动对不同人群血压、心率变异的影响[J].北京体育大学 学报,2011,34(12):59-62.

[8] 黄莹仪.太极拳对大学生心理健康的影响[J].科技风，2010:18.

[9] 魏晓磊，梁爽.吉林省教育学院学报[J].2012,28(04):106-107.

[10] Karen Caldwell,Lisa Emery,Mandy Harrison,Jeffrey Greeson. Changes in Mindfulness, Well-Being, and Sleep Quality in College Students Through Taijiquan Courses:A Cohort Control Study[J]. The Journal Of Alternative and Complementary Medcine,2011,17(10):931-938.

[11] Janisse HC, Nedd D, Escamilla S, Nies MA: Physical activity, social support, and family structure as determinants of mood among European-American and African-American women. Women Health 2004,39:101-116.

[12] Chow YW,Tsang HW: Biopsychosocial effects of qigong as a mindful exercise for people with anxiety disorders: a speculative review.[J].Altern Complement Med 2007,13:831-839.

[13] Smith PJ, Blumenthal JA, Babyak MA, Georgiades A, Hinderliter A, Sherwood A: Effects of exercise and weight loss on depressive symptoms among men and women with hypertension.J Psychosom Res 2007, 63:463-469.

[14] Broman-Fulks JJ, Storey KM: Evaluation of a brief aerobic exercise intervention for high anxiety sensitivity. Anxiety, Stress &amp; Coping 2008, 21:117-128.

[15]Tsang HW, Chan EP, Cheung WM: Effects of mindful and non-mindful exercises on people with depression: a systematic review. Br J Clin Psychol 2008, 47:303-322.
